# Ailanthone Inhibits Huh7 Cancer Cell Growth *via* Cell Cycle Arrest and Apoptosis *In Vitro* and *In Vivo*

**DOI:** 10.1038/srep16185

**Published:** 2015-11-03

**Authors:** Zhenjian Zhuo, Jianyang Hu, Xiaolin Yang, Minfen Chen, Xueping Lei, Lijuan Deng, Nan Yao, Qunlong Peng, Zhesheng Chen, Wencai Ye, Dongmei Zhang

**Affiliations:** 1 Guangdong Province Key Laboratory of Pharmacodynamic Constituents of TCM and New Drug Research, College of Pharmacy, Jinan University, Guangzhou 510632, China; 2 Department of Pharmaceutical Sciences, College of Pharmacy and Health Sciences, St. John’s University, Queens, New York, United States

## Abstract

While searching for natural anti-hepatocellular carcinoma (HCC) components in *Ailanthus altissima*, we discovered that ailanthone had potent antineoplastic activity against HCC. However, the molecular mechanisms underlying the antitumor effect of ailanthone on HCC have not been examined. In this study, the antitumor activity and the underlying mechanisms of ailanthone were evaluated *in vitro* and *in vivo*. Mechanistic studies showed that ailanthone induced G_0_/G_1_-phase cell cycle arrest, as indicated by decreased expression of cyclins and CDKs and increased expression of p21 and p27. Our results demonstrated that ailanthone triggered DNA damage characterized by activation of the ATM/ATR pathway. Moreover, ailanthone-induced cell death was associated with apoptosis, as evidenced by an increased ratio of cells in the subG_1_ phase and by PARP cleavage and caspase activation. Ailanthone-induced apoptosis was mitochondrion-mediated and involved the PI3K/AKT signaling pathway in Huh7 cells. *In vivo* studies demonstrated that ailanthone inhibited the growth and angiogenesis of tumor xenografts without significant secondary adverse effects, indicating its safety for treating HCC. In conclusion, our study is the first to report the efficacy of ailanthone against Huh7 cells and to elucidate its underlying molecular mechanisms. These findings suggest that ailanthone is a potential agent for the treatment of liver cancer.

Primary hepatic cancer or liver cancer is the fifth-most-common global malignancy and the third-most-common cause of cancer-related death^1^. The most frequent hepatic cancer is hepatocellular carcinoma (HCC), which accounts for approximately 75% of all primary liver cancers and causes more than 600,000 deaths each year^2^. HCC is an aggressive malignancy that is frequently diagnosed at a stage when it has already metastasized or is locally advanced^3^.

Currently, therapeutic strategies for HCC include surgery, radiation and chemotherapy^4^. However, despite advances in multimodal treatments, no effective systemic therapy has been established for advanced HCC after the failure of first-line therapy with sorafenib^5^,^6^. The overall 5-year survival rate for patients with liver cancer after receiving systemic treatment is generally less than 14%^7^. Chemotherapy is an important therapeutic approach for the treatment of advanced hepatocellular carcinoma, but its clinical applications are limited due to its severe side effects^8^,^9^. Therefore, identifying novel, effective therapeutic agents with low toxicity remains a major task for the treatment of HCC. Natural products serve as a significant source of anticancer agents; indeed, over 60% of anticancer drugs are derived from natural compounds^10^. Therefore, identifying naturally occurring agents is a promising approach for anticancer treatment.

Ailanthone, one of the primary potent quassinoids, is an important active compound isolated from the well-known traditional Chinese medicinal herb *Ailanthus altissima*^11^. Ailanthone has a wide spectrum of biological activities, including anti-inflammatory, anti-HIV, anti-malarial, anti-allergic, antiulcer and antimicrobial activities^12^,^13^. Ailanthone has been shown to possess an *in vitro* growth-inhibitory effect against several cancer cell lines, including HepG2, Hep3B, R-HepG2, Jurkat, HeLa, MCF-7, MDA-MB-231 and A549 cells^14^,^15^,^16^,^17^. Rosati *et al.* found that ailanthone could induce mitochondrial membrane depolarization and caspase-3 activation in Jurkat cancer cells^14^. However, neither the *in vitro* and *in vivo* efficacy of ailanthone against Huh7 liver cancer cells nor its safety has yet been reported.

In the present study, we sought to investigate the cytotoxic effects of ailanthone on the Huh7 HCC cell line and to elucidate its underlying mechanisms *in vitro* and *in vivo*. Here, we report for the first time that ailanthone inhibits Huh7 cell growth through the induction of mitochondrion-mediated cell apoptosis and G_0_/G_1_ cell cycle arrest. We also found that ailanthone significantly inhibited tumor growth *in vivo* without marked toxicity. These findings provide strong evidence that ailanthone may has the potential to be used for the clinical treatment of HCC.

## Results

### Ailanthone inhibits the growth of hepatic cancer cells *in vitro*

**Figure 1:**
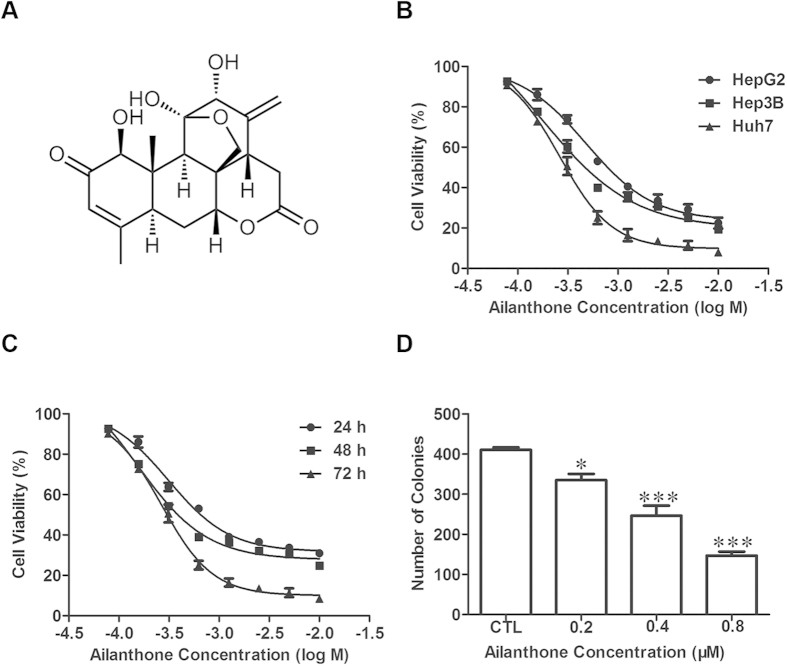
Ailanthone treatment inhibits the viability of Huh7 cells *in vitro*. (**A**) Chemical structure of ailanthone isolated from *Ailanthus altissima*. (**B**) The viability of HepG2, Hep3B and Huh7 cells treated with ailanthone at the indicated concentrations for 72 h was determined using an MTT assay. (**C**) The viability of Huh7 cells treated with ailanthone at the indicated concentrations for 24 h, 48 h and 72 h was determined using an MTT assay. (**D**) The clonogenicity of Huh7 cells after treatment with ailanthone was measured with a colony-formation assay. The data are expressed as the mean ± SD of 3 independent experiments. *P < 0.05 and ***P < 0.001 compared with the control.

The inhibitory effect of ailanthone on 3 human hepatic cell lines (HepG2, Hep3B and Huh7) was measured with the MTT assay. Ailanthone potently inhibited the growth of HepG2, Hep3B and Huh7 cells in a concentration-dependent manner (Fig. 1B), with IC_50_ values of 0.628 ± 0.047, 0.544 ± 0.031 and 0.350 ± 0.016 μM, respectively, after 72 h of treatment. Because ailanthone showed the most potent cytotoxic effect against Huh7 cells, its cytotoxic effect against this cell line was further evaluated. As shown in Fig. 1C, treatment with ailanthone reduced the viability of Huh7 cells in a concentration- and time-dependent manner, with IC_50_ values of 0.677 ± 0.050, 0.399 ± 0.015 and 0.351 ± 0.017 μM after 24 h, 48 h and 72 h of treatment, respectively. A colony-formation assay was used to evaluate the long-term effect of ailanthone on cell survival. The inhibitory effect of ailanthone on colony formation was also concentration-dependent (Fig. 1D), which confirmed the cytotoxic effect of ailanthone against Huh7 cells.

**Figure 2:**
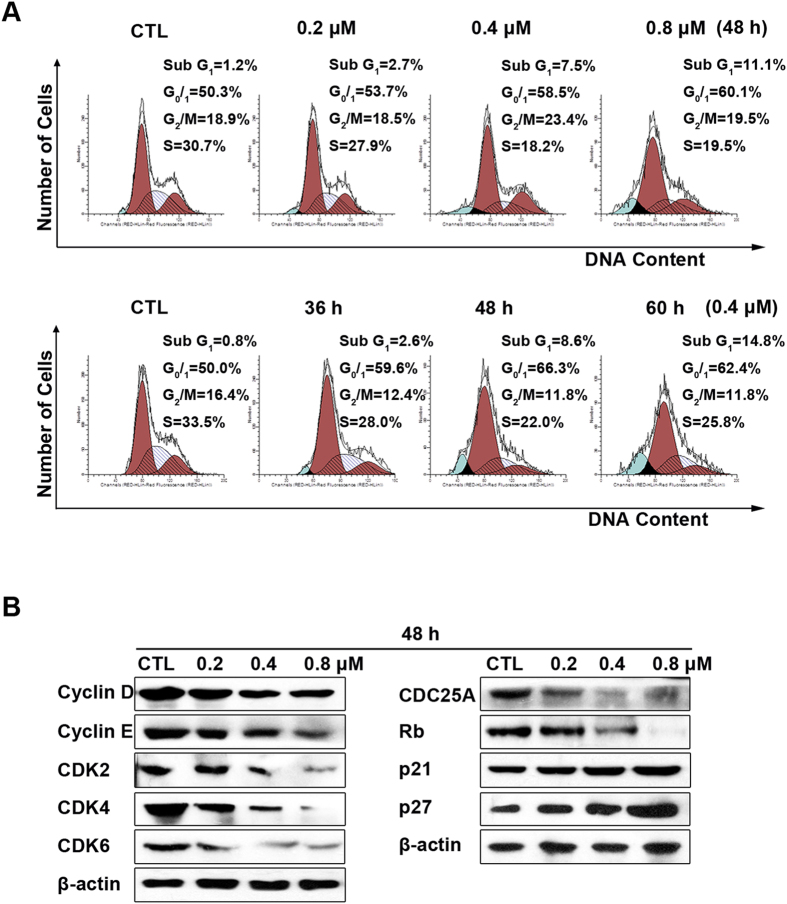
Ailanthone induces G_0_/G_1_ cell cycle arrest in Huh7 cells. (**A**) Cell cycle profiles were measured by flow cytometry following treatment of the cells with various concentrations of ailanthone (0, 0.2, 0.4 or 0.8 μM) for 48 h or for various times (0, 36 h, 48 h, or 60 h) with 0.4 μM ailanthone. (**B**) Huh7 cells were treated with various concentrations of ailanthone or for various times. Western blotting was performed to detect p21, p27, cyclin D, cyclin E, CDK2, CDK4, CDK6, CDC25A and Rb. β-actin was used as a loading control.

**Ailanthone induces cell cycle arrest by regulating cell cycle regulatory proteins.** To investigate whether the anti-proliferative effect of ailanthone on Huh7 cells was triggered by cell cycle arrest, we measured the cell cycle phase ratio by flow cytometry with propidium iodide (PI) staining. As shown in Fig. 2A, ailanthone treatment induced significant G_0_/G_1_-phase accumulation compared with the control. The percentage of G_0_/G_1_ cells increased significantly with ailanthone concentration, from 50.3% to 60.1%, and with time, from 50.0% to 62.4%. To investigate the molecular basis by which ailanthone inhibited the G_0_/G_1_ transition in tumor cells, we treated cells with ailanthone and then analyzed the expression of proteins involved in cell cycle regulation. We found that ailanthone treatment inhibited cyclin D and cyclin E expression and reduced the expression of CDK2, CDK4 and CDK6; in contrast, p21 and p27 were increased in Huh7 cells (Fig. 2B). The expression level of cell division cycle 25A (CDC25A), which acts as an upstream regulator of the CDK/cyclin complex, was significantly inhibited by ailanthone. Ailanthone treatment also reduced the expression of the retinoblastoma protein (Rb), which is a positive regulator of the cell cycle.

### Ailanthone induces DNA damage and activates the sensor kinases ATM and ATR

**Figure 3:**
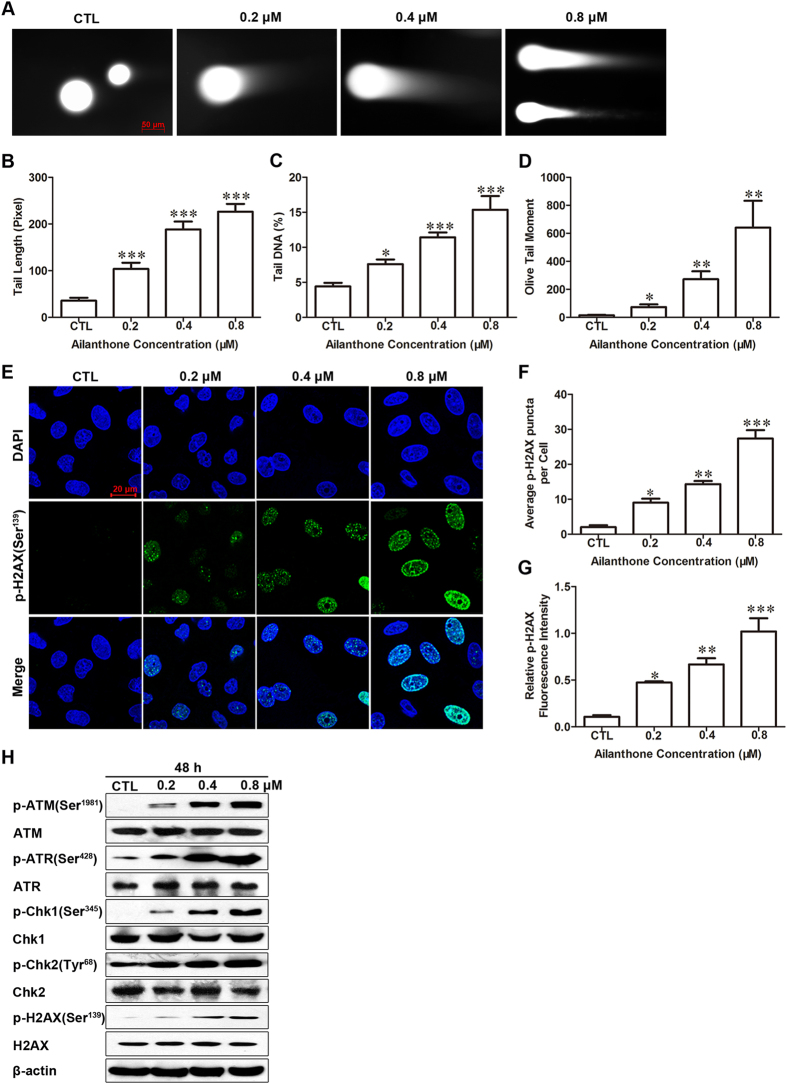
Ailanthone induces DNA damage and triggers activation of the ATM/ATR pathway in Huh7 cells. (**A**). Representative comet images of alkaline gel electrophoresis demonstrating DNA damage in Huh7 cells (original magnification: 200×). Huh7 cells were evaluated with a comet assay after treatment with ailanthone. DNA migration was quantified by surveying tail length (**B**), tail DNA% (**C**) and olive tail moment (**D**) using MetaXpress software. At least 10 cells were evaluated per sample. The results are presented as the mean ± SD. *P < 0.05, **P < 0.01 and ***P < 0.001 compared with the control. (**E**) Representative Immunofluorescence images of p-H2AX (Ser^139^). After treatment with various concentrations of ailanthone for 48 h, the cells were incubated with antibodies against p-H2AX (Ser^139^) and then with an Alexa Fluor 488-conjugated secondary antibody and DAPI. Images were obtained using a laser scanning confocal microscope (original magnification: 400×). (**F**) Graph representing the average number of p-H2AX puncta per cell. (**G**) Graph representing the relative fluorescence intensity of p-H2AX. The data are expressed as the mean ± SD in triplicate from one experiment. *P < 0.05, **P < 0.01 and ***P < 0.001 compared with the control. (**H**) The levels of phosphorylated and total ATM, ATR, Chk1, Chk2, and H2AX were assessed by western blot. β-actin was used as a loading control.

Cells with damaged DNA that is difficult to repair may undergo cell cycle arrest at the G_0_/G_1_ phase^18^. We applied the comet assay to study whether ailanthone induced DNA damage, as this assay allows the rapid and sensitive detection of a broad spectrum of DNA damage. Treatment with ailanthone resulted in increased comet parameters such as tail length, tail DNA (%) and olive DNA moments (Fig. 3A–D), thereby indicating that the level of DNA damage increased in a concentration-dependent manner.

Phosphorylation of histone H2AX (Ser^139^) is a sensitive molecular marker for double-stranded DNA breaks (DSBs)^19^. Immunofluorescence images of phosphorylated histone H2AX (p-H2AX) showed that ailanthone caused a concentration-dependent induction of p-H2AX foci; this finding was further confirmed by western blot analysis (Fig. 3E–H).

To elucidate whether the DNA damage induced by ailanthone led to activation of the ATM/ATR DNA damage signaling pathway, the levels of phosphorylated and total ATM and ATR and of their target molecules (i.e., Chk1 and Chk2) were determined in Huh7 cells. The results showed that ailanthone treatment resulted in rapid phosphorylation of ATM and ATR at their Ser^1981^ and Ser^428^ sites, respectively. Chk1/2, which are key effectors of cell cycle checkpoint protein kinases and are phosphorylated by ATM/ATR to initiate the checkpoint-cascade pathway, were activated, as indicated by the increase in Chk1/2 phosphorylation after ailanthone treatment (Fig. 3H). These results indicated that ailanthone could induce DNA DSBs in Huh7 cells and activate the ATM/ATR and Chk1/Chk2 pathways; these effects may trigger the arrest of cell-cycle progression at the G_0_/G_1_ phase.

### Ailanthone induces caspase-dependent apoptosis

**Figure 4:**
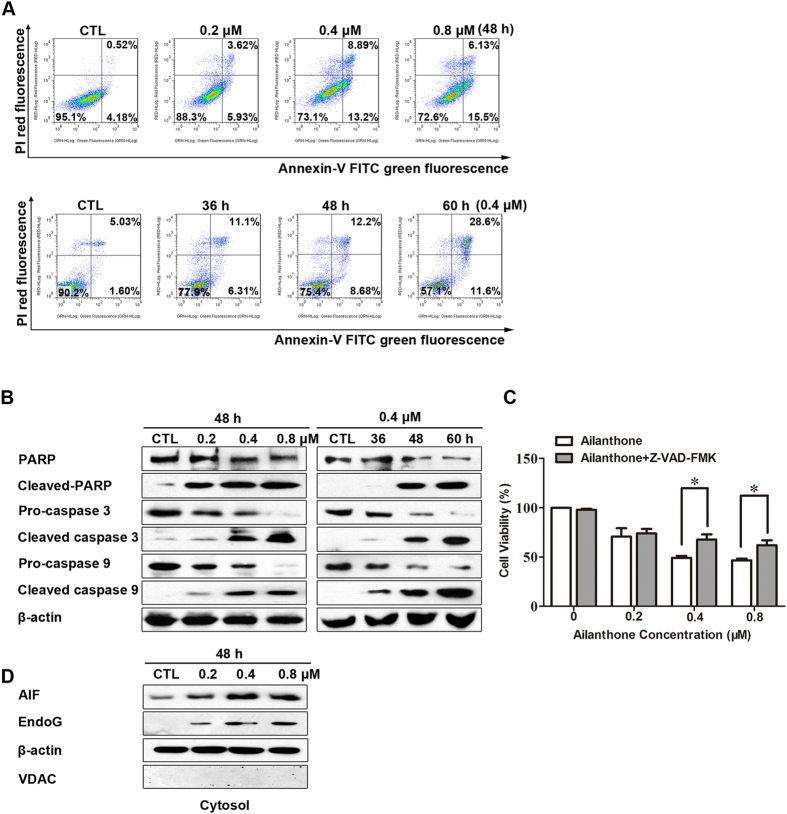
Ailanthone induces caspase-dependent and caspase-independent apoptosis in Huh7 cells. (**A**) Flow cytometric analysis of ailanthone-induced apoptosis in Huh7 cells using Annexin V-FITC/PI staining. Cells in the lower right quadrant (Annexin V^+^/PI^−^) represent early apoptotic cells, and those in the upper right quadrant (Annexin V^+^/PI^+^) represent late apoptotic cells. (**B**) Ailanthone activates caspase cleavage in a concentration- and time-dependent manner. Equal amounts of whole-cell extracts were separated by 10% SDS-PAGE, electrotransferred onto PVDF membranes, and analyzed by western blotting using the indicated antibodies against proteins related to caspase-dependent apoptosis. β-actin was used as a loading control. (**C**) Cells were pre-treated with the indicated concentration of z-VAD-fmk (20 μM) for 1 h prior to treatment. Cell viability was determined using MTT. The data are expressed as the mean ± SD of 3 independent experiments. ***P < 0.001 compared with the control group. (**D**) Huh7 cells were treated with various concentrations of ailanthone for 48 h. Western blotting was performed to detect the cytosolic levels of AIF and EndoG. β-actin and VDAC were used as the loading controls.

The above data showed that ailanthone treatment led to the accumulation of cells in the subG_1_ phase (Fig. 2A), indicating the occurrence of apoptosis. To confirm the occurrence of apoptosis, we performed an Annexin V-FITC/PI double-staining assay. As shown in Fig. 4A, the percentage of apoptotic cells (including early and late apoptotic cells) increased significantly with ailanthone concentration, from 4.7% to 21.6%, and with time, from 6.6% to 40.2%. Because caspase activation is considered to be a hallmark of apoptosis^20^, we performed western blotting to examine caspase activation. Cleaved caspase-9 and cleaved caspase-3 were clearly increased in ailanthone-treated cells. Cleaved PARP was also strongly activated in ailanthone-treated Huh7 cells (Fig. 4B).

To evaluate the function of caspases in cell apoptosis, Huh7 cells were pretreated with the pan-caspase inhibitor z-VAD-fmk for 1 h prior to treatment. As shown in Fig. 4C, z-VAD-fmk pretreatment significantly reduced cell death. These results confirm that ailanthone may promote apoptosis *via* a caspase-dependent mechanism.

Ailanthone-induced apoptosis in Huh7 cells may be also partially caspase-independent since z-VAD-fmk did not completely block apoptosis. Apoptosis-inducing factor (AIF) and endonuclease G (EndoG) are caspase-independent apoptotic factors released into the cytosol, which can be translocated to the nucleus and then cleave chromatin DNA independently of caspases^21^. To determine whether AIF and EndoG were involved in ailanthone-induced apoptosis of Huh7 cells, the cytosolic levels of AIF and EndoG were analyzed. As shown in Fig. 4D, Western blot analysis showed that ailanthone upregulated the cytosolic levels of AIF and EndoG in Huh7 cells.

### Ailanthone induces apoptosis through mitochondrial pathways

**Figure 5:**
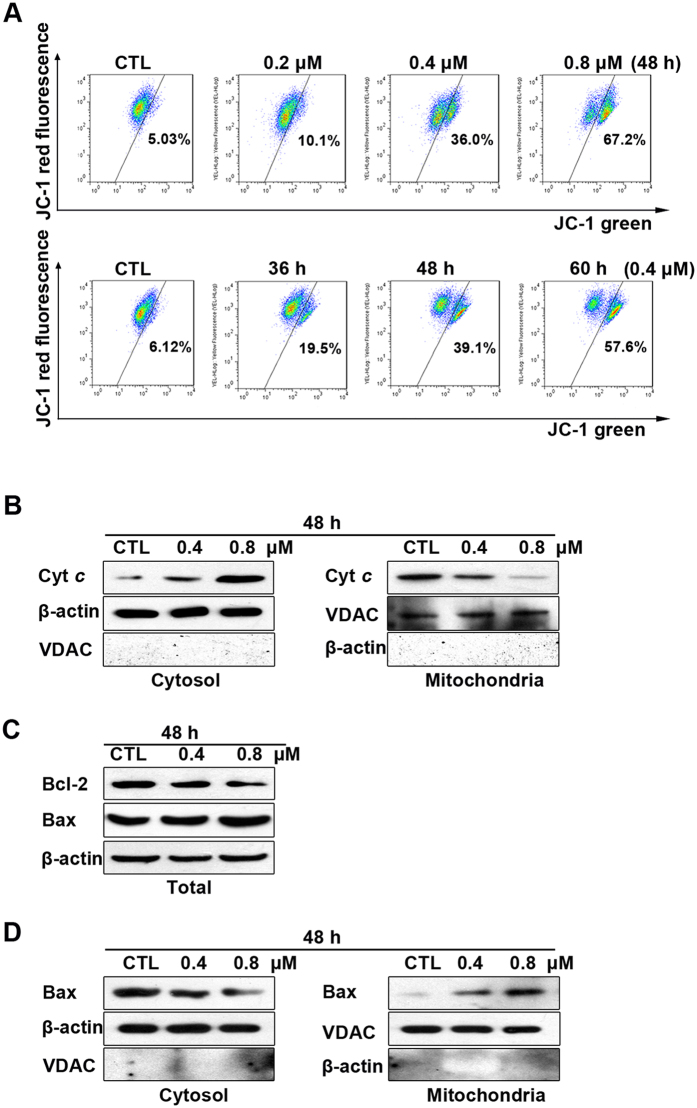
Ailanthone induces apoptosis *via* the mitochondrial pathway in Huh7 cells. (**A**) Decrease in mitochondrial potential induced by ailanthone. After treatment with ailanthone, the cells were stained with JC-1 for 15 min and analyzed by flow cytometry. Changes in Δ*Ψm* were detected by the ratio of red fluorescence (JC-1 aggregates) to green fluorescence (JC-1 monomers). (**B**) The cytosolic and mitochondrial levels of the pro-apoptotic proteins cytochrome *c* in Huh7 cells treated with or without ailanthone were assessed by western blotting. (**C**) The total expression of Bcl-2 and Bax in Huh7 cells treated with or without ailanthone was assessed by western blotting. (**D**) Huh7 cells were treated with various concentrations of ailanthone for different amounts of time. Western blotting was performed to detect Bax in the cytosol and mitochondria. β-actin and the voltage-dependent anion channel (VDAC) were used as the loading controls for the cytosol and mitochondrial fractions.

The mitochondrion-dependent pathway is the most common apoptotic pathway in vertebrate tumor cells^22^. To investigate the mechanism underlying ailanthone-induced Huh7 cell apoptosis, we measured the loss of mitochondrial transmembrane potential (ΔΨm) using JC-1. As shown in Fig. 5A, the green fluorescence of the JC-1 monomers increased from 5.03% to 67.2% and from 6.12% to 57.6% after treatment with various concentrations of ailanthone for various times, suggesting that ailanthone induced a loss of mitochondrial membrane potential in Huh7 cells in a concentration- and time-dependent manner.

Mitochondrial dysfunction, as indicated by the dissipation of Δ*Ψm*, could subsequently cause the release of cytochrome *c* from mitochondria into the cytosol^22^. Therefore, we evaluated the cytosolic and mitochondrial cytochrome *c* level. Western blot analysis showed that treatment with ailanthone for 48 h resulted in a striking increase in the level of cytosolic cytochrome *c* and a decrease in the level of mitochondrial cytochrome *c* compared with untreated cells (Fig. 5B). Next, we investigated the expression levels of the Bcl-2 family of apoptosis regulator proteins. As shown in Fig. 5C, Bcl-2 was decreased, whereas Bax was increased. Moreover, the translocation of Bax from the cytosol to the mitochondria was also increased by ailanthone treatment (Fig. 5D).

### Ailanthone inhibits PI3K/AKT in Huh7 cells

**Figure 6:**
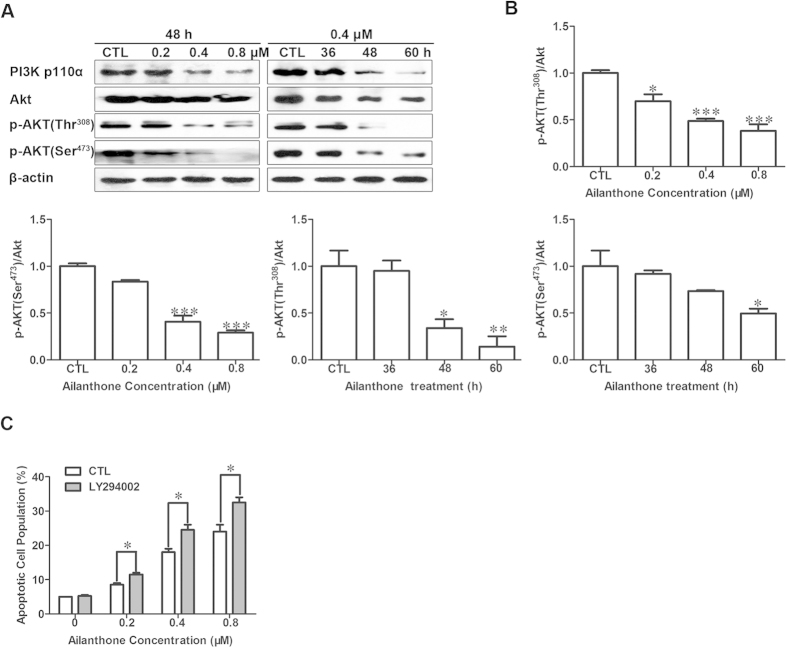
Ailanthone induces apoptosis through Akt inactivation in Huh7 cells. (**A**) Total cell lysates were prepared and subjected to SDS-PAGE followed by western blot analysis to measure the protein levels of a subunit of PI3K (p110α) and of p-Akt (Thr^308^ and Ser^473^). β-actin was used as the protein loading control. The immunoblots shown are representative of 3 independent experiments. (**B**) Densitometric analysis of phosphoAkt-to-total Akt ratios was shown. *P < 0.05, **P < 0.01 and ***P < 0.001 compared with the control. (**C**)Effect of LY294002 on ailanthone-induced apoptosis. Cells were treated with LY294002 (4 mM) for 1 h prior to ailanthone treatment. The percentage of apoptotic cells after ailanthone treatment in the presence or absence of LY294002 was detected by staining with Annexin V-FITC/PI and analyzed by flow cytometry. The data are shown as the mean ± SD of 3 independent experiments. *P < 0.05 compared with ailanthone alone.

Because the PI3K/AKT pathway acts as a key regulator of cellular survival and apoptosis in a broad variety of human cancers, we examined the effects of ailanthone on the PI3K/AKT pathway and evaluated whether PI3K/AKT played a central role in ailanthone-mediated apoptotic cell death. As shown in Fig. 6A,B, ailanthone treatment decreased the levels of the catalytic (p110α) subunit of PI3K and the levels of Akt and p-Akt (Thr^308^ and Ser^473^) in a concentration- and time-dependent manner. To further investigate the role of Akt in ailanthone-induced apoptotic cell death, we used the Akt inhibitor LY294002. Inactivation of Akt by LY294002 markedly increased ailanthone-induced apoptotic cell death (Fig. 6C). Taken together, these results demonstrate that the PI3K/AKT pathway is inactivated by ailanthone and plays a key role in regulating ailanthone-mediated apoptosis.

### Ailanthone inhibits the growth and angiogenesis of Huh7-cell xenografts in nude mice

**Figure 7:**
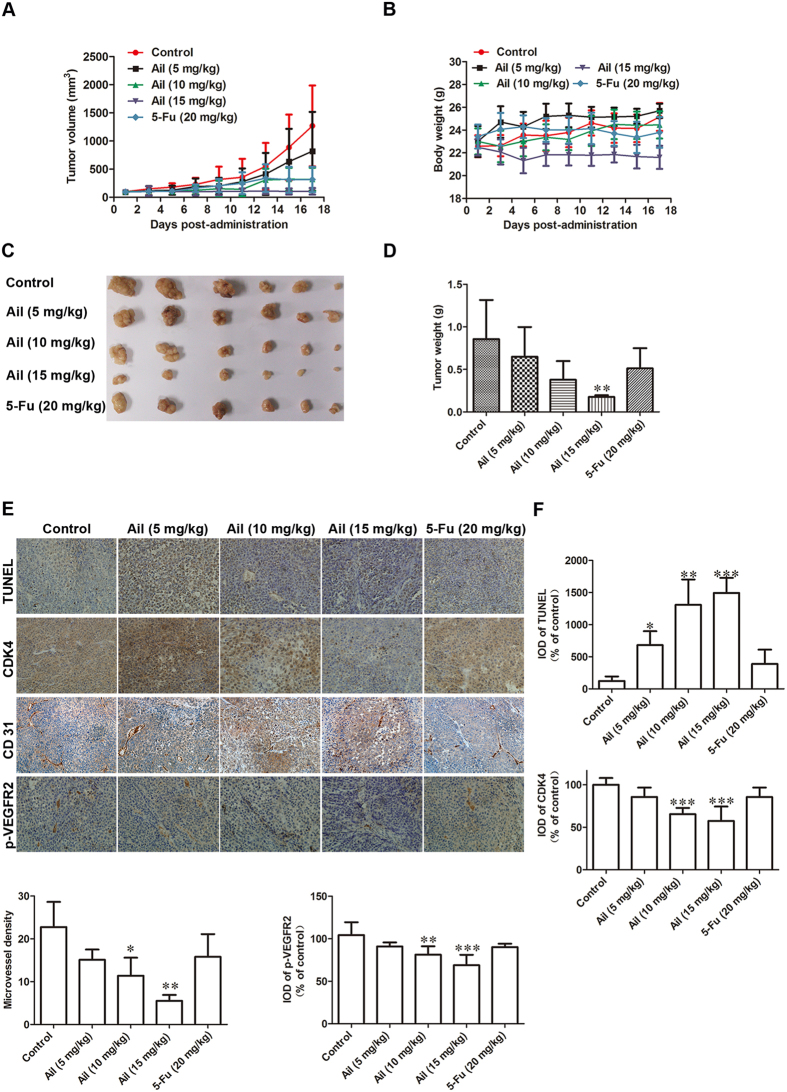
Ailanthone inhibits tumor growth and angiogenesis in a nude mouse Huh7 tumor xenograft model. After tumor development, mice were randomly placed into five groups (n = 6 per group) and treated daily with 5 mg/kg, 10 mg/kg, or 15 mg/kg of ailanthone, 20 mg/kg of 5-Fu, or saline by intraperitoneal injection for 16 days. (**A**) Tumor volume, (**B**) body weight, (**C**) tumors and (**D**) tumor weight were measured. Representative images of immunohistochemical detection of (**E**) TUNEL, CDK4, CD31, and p-VEGFR2 in tumor tissues were detected using immunohistochemistry (400×). (**F**) The quantitative results of TUNEL, CDK4, CD31, and p-VEGFR2. The data are shown as the mean ± SD from 6 individual mice (n = 6) in each group from one experiment. *P < 0.05, **P < 0.01 and ***P < 0.001 compared with the controls.

Next, we investigated whether ailanthone could prevent Huh7 progression *in vivo*. Nude mice were subcutaneously injected in the right flank with Huh7 cells. The Huh7-bearing mice were randomly divided into 5 groups (6 mice per group) and treated with vehicle, 5-fluorouridine (5-Fu) or ailanthone at a dose of 5 mg/kg, 10 mg/kg, or 15 mg/kg daily for 16 days. As shown in Fig. 7A, the tumor volume in ailanthone-treated mice at doses of 5 mg/kg, 10 mg/kg and 15 mg/kg per day was significantly reduced compared to the control (*P* < 0.05). The mice in the 5 mg/kg and 10 mg/kg treatment groups maintained normal weight gain throughout the treatment, while the 15 mg/kg dose mildly affected normal weight (Fig. 7B). The tumors were harvested at the time of euthanasia, and the wet weights of the tumor and mouse in each treatment group were recorded. As shown in Fig. 7C,D, the wet weights of the tumors were significantly lower in mice in the treated groups when compared to the control group (*P* < 0.05).

Next, we evaluated whether ailanthone inhibited xenograft tumor growth by inducing cell apoptosis and cell cycle G_0_/G_1_ arrest *in vivo*. We observed an increase in the cell apoptosis marker TUNEL (Fig. 7E,F). Moreover, the expression levels of the cell cycle G_0_/G_1_ regulatory protein CDK4 was significantly lower in tumor xenografts from treated mice than in tumor xenografts from control mice (Fig. 7E,F). To determine the effect of ailanthone on angiogenesis *in vivo*, we performed immunohistochemical staining to detect the expression of the endothelial cell-specific marker CD31 and thereby to examine intratumoral microvessel density (MVD). The density of CD31-positive vessels was significantly decreased in the ailanthone-treated tumors (Fig. 7E,F). Ailanthone also resulted in a marked reduction of p-VEGFR2 level in solid tumors (Fig. 7E,F), suggesting that disruption of vascularity may underlie the tumor-suppressive effect of ailanthone.

### Ailanthone does not cause obvious toxicity in nude mice

**Figure 8:**
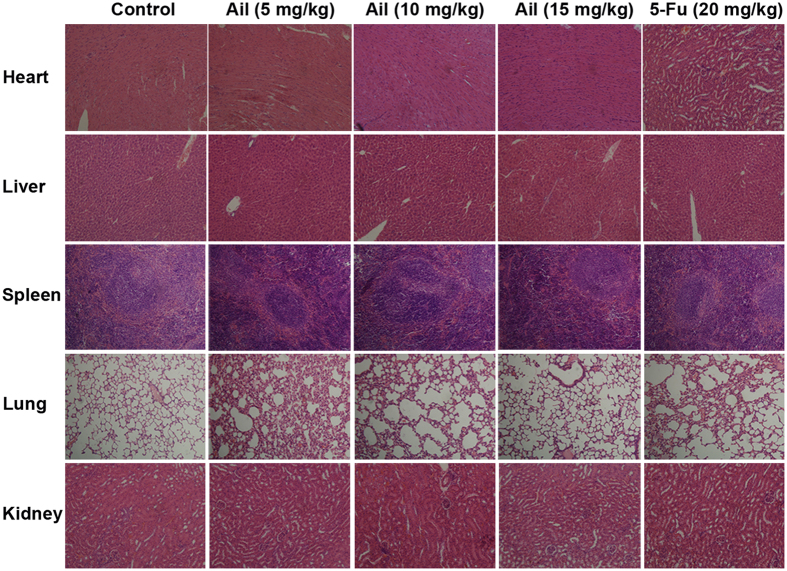
Ailanthone shows no obvious toxicity to nude mice. Histological analysis of heart, lung, liver, kidney, and spleen in HCC xenograft mice. After the mice were euthanized, the organs were excised and stained with hematoxylin and eosin (H&E). The images of each group are depicted at an original magnification of 200×. Representative sections of Huh7 HCC xenografts are shown.

To examine the *in vivo* cytotoxic effects of ailanthone, we evaluated sub-acute toxicity by performing hematoxylin-eosin (H&E) staining in tumor tissues excised from the mice. Pathological screening of H&E-stained sections of heart, lung, liver, kidney, and spleen revealed no apparent changes in the ailanthone-treated animals (Fig. 8).

## Discussion

The Chinese herbal medicine *Ailanthus altissima* has been shown to possess potent anticancer activities^23^. Ailanthone, one of the primary active quassinoids in *Ailanthus altissima*, has also been reported to possess certain anticancer properties^17^. However, the antitumor activity of ailanthone against human liver cancer and its mechanism of action have not been elucidated. Therefore, the purpose of this study is to examine the effect of ailanthone on human HCC and to elucidate the underlying molecular mechanisms. The present study shows for the first time that ailanthone inhibits the proliferation of Huh7 cells *in vitro* and *in vivo*. We also revealed the molecular mechanisms by which ailanthone affects Huh7 cells.

Our results showed that ailanthone suppressed the growth of Huh7 cells in a concentration- and time-dependent manner. Deregulation of cell cycle progression is a common feature of cancer^24^. Therefore, targeting the regulatory components of the cell cycle machinery has been proposed as an important strategy for the treatment of human malignancies. Diverse natural compounds inhibit cancer cell growth by arresting the cell cycle^25^,^26^. We found that ailanthone-mediated G_0_/G_1_ cell cycle arrest contributed to the inhibition of proliferation in Huh7 cells. The cell cycle is regulated by multifaceted proteins that primarily include two classes of molecules: the cyclin-dependent kinases (Cdks), a family of serine/threonine kinases, and their cyclin binding partners^27^. Cyclin D and cyclin E (along with CDK2, CDK4, and CDK6) play important roles in the progression of cells through the G_1_ phase of the cell cycle^28^. This fact prompted us to study the expression profiles of cyclins in the present study. Ailanthone induced cell cycle arrest by downregulating the expression levels of cyclin D/cyclin E and CDK2/CDK4/CDK6, suggesting that these proteins are involved in cell cycle progression in Huh7 cells. Ailanthone-induced G_0_/G_1_ phase cell cycle arrest was confirmed by the downregulation of CDC25A and Rb, which control progression through the G_1_ phase of the cell cycle^29^. The Cip/Kip family proteins p21 and p27 are potent cyclin-dependent kinase inhibitors that bind to and inhibit the activities of Cdks; thus, these proteins function as regulators of cell cycle progression through the G_1_ and S phases. Increased levels of these proteins thus indicate the induction of G_0_/G_1_ cell cycle arrest^30^. The protein levels of p21 and p27 were increased in ailanthone-treated Huh7 cells, indicating that ailanthone treatment is related to p21- and p27-dependent cell cycle arrest. In summary, these results clearly indicate that ailanthone modulates G_1_-phase proteins, resulting in the arrest of Huh7 cells.

Cell cycle arrest can be triggered by various stimulating factors and may result in the blockage of cell division, cell death, and/or apoptosis. In eukaryotic cells, cell cycle arrest is mainly regulated by the DNA damage checkpoint pathway^31^. Additionally, caspase activation is accompanied by the activation of PARP, which indicates the activation of the DNA repair mechanism. To further explore the mechanisms underlying the regulation of the cell cycle by ailanthone, we applied the comet assay because it is a rapid and sensitive method for the detection in individual cells of DNA damage induced by a variety of chemical agents. Our studies showed that ailanthone induced long tails in the comet assay. The formation of aggregates in the nucleus due to phosphorylation of histone −H2AX is a marker of DNA double-strand breaks; thus, the measurement of p-H2AX (Ser^139^) focus formation is a sensitive method for the detection of DNA DSBs^32^. The increase in p-H2AX (Ser^139^) focus formation indicated that ailanthone increased DNA damage in a concentration-dependent manner. The upregulation of p-H2AX (Ser^139^) level in Huh7 cells as revealed by western blotting further confirmed the presence of DNA damage.

ATM and ATR are members of the phosphatidylinositol-3-kinase-related (PIKK) family and can be activated in response to many different types of DNA damage^33^. In this study, we found that ATM and ATR were activated after treatment with ailanthone. Generally, ATM phosphorylates the downstream effector Chk2 and ATR phosphorylates the downstream effector Chk1. Next, the phosphorylated effectors downregulate the expression of CDC25A, thereby inhibiting the CDK2/cyclin E and CDK4/6/cyclin D kinases and subsequently leading to cell cycle arrest at the G_0_/G_1_ phase^34^. In our study, we observed a significant increase in Chk1 phosphorylation on Ser^345^ and Chk2 on Tyr^68^ after ailanthone exposure. Taken together, the results demonstrate that ailanthone may induce DNA damage and G_1_ checkpoint arrest in Huh7 cells through the ATM/ATR-Chk1/Chk2 signaling pathway.

In addition to delaying cell cycle progression, DNA damage is also associated with mitochondrial apoptotic pathway-triggered apoptosis^35^. Mitochondria play an essential role in cell death signal transduction^36^. The ailanthone-induced activation of caspases 9 and 3 in Huh7 cells suggested that mitochondria were responsible for the apoptotic potential of ailanthone. Mitochondrial involvement was confirmed by the collapse of the mitochondrial transmembrane potential (Δ*Ψm*) in ailanthone-treated cells. Mitochondrial apoptotic proteins such as the independent-caspase cascades AIF and EndoG were increased in the cytosol in Huh7 cells, indicating that AIF and EndoG might be involved in ailanthone-induced apoptosis. Members of the Bcl-2 family of proteins play key roles in controlling the mitochondrial pathway^37^,^38^. The Bcl-2 family can generally be classified into two subgroups: those that promote cell death (e.g., Bax) and those involved in survival (e.g., Bcl-2). We found that treatment with ailanthone resulted in a decrease in the expression of the Bcl-2 protein in Huh7 cells and an associated elevation in the levels of the Bax protein; moreover, Bax was translocated from the cytosol to the mitochondria in a dose-dependent manner. Mitochondrial involvement was further demonstrated by the observed decrease in the expression of mitochondrial cytochrome *c* in Huh7 cells. Nevertheless, the cell death processes induced by ailanthone are very complicated. We will further investigate the involvement of other manners of cell death in ailanthone-treated cells, such as necrosis and autophagy.

Genetic abnormalities in the phosphatidylinositol-3-kinase (PI3K)/AKT pathway are common in human cancer, and there is increasing evidence that PI3K/AKT is involved in the development of many types of cancers^39^,^40^. The significance of the PI3K/AKT pathway and its potential as a therapeutic target for cancer treatment have been investigated in preclinical studies of several types of human cancer, including renal cancer, lung cancer, breast cancer, glioblastoma, neuroblastoma, and HCC. The results of these studies suggest that PI3K/AKT and its downstream pathways are promising targets for therapeutic intervention^41^,^42^. Because the PI3K/AKT pathway is known to play an important role in cell cycle progression, apoptosis and tumorigenesis, we hypothesized that the PI3K/AKT pathway might also play a role in ailanthone-induced apoptosis. We demonstrated that treatment of Huh7 cells with ailanthone resulted in decreases both of the expression of PI3K (p110α) and of the phosphorylation of Akt at Ser^473^ and Thr^408^. Combined with the results obtained by treating Huh7 cells with ailanthone and the Akt inhibitor LY294002, we verified that ailanthone-induced apoptosis could at least in part be attributed to Akt inactivation.

To determine the potential of ailanthone to inhibit tumor growth *in vivo*, we evaluated the anti-tumor activity of ailanthone against Huh7 tumors in a nude mouse xenograft model. The results showed that ailanthone inhibited tumor growth *in vivo* in a dose-dependent manner at a well-tolerated dose. The mean tumor volumes and tumor weights of the ailanthone treatment groups were significantly lower than in the control group. At present, no major toxicities have been noted in animals treated with ailanthone.

To determine whether the mechanisms underlying tumor growth inhibition by ailanthone *in vivo* were consistent with the *in vitro* results, we used immunohistochemistry to examine the expression of molecules associated with cell proliferation and G_0_/G_1_ cell cycle arrest in the xenografts. Indeed, tumor growth inhibition by ailanthone was associated with G_0_/G_1_ cell cycle arrest and apoptosis *in vivo*.

Angiogenesis is essential for the development of a blood supply that can provide sufficient oxygen and nutrients to cancer cells and thereby promote solid tumor growth and metastasis^43^. Without blood vessels, tumors cannot grow beyond a critical size or metastasize to other tissues^44^. Based on its potent inhibition of tumor growth, we hypothesized that ailanthone might reduce tumor vascularization. We found a decrease in the angiogenesis marker CD31 and p-VEGFR2 in the tissues of mice treated with ailanthone compared with the control mice. This result indicates that ailanthone suppresses tumor growth in part by disrupting tumor vascularity. However, because angiogenesis is a dynamic and complicated process regulated by multiple pathways^45^, the functional role of ailanthone in angiogenesis and its underlying mechanism should be further investigated in future studies.

In conclusion, we demonstrated that ailanthone from *Ailanthus altissima* has a growth-inhibitory effect on HCC cells both *in vitro* and *in vivo*. We revealed that ailanthone induces tumor cell apoptosis through the caspase-dependent mitochondrial pathway and *via* G_0_/G_1_ phase arrest by regulating G_0_/G_1_ checkpoint proteins and the DNA damage response-signaling pathway. Additionally, ailanthone induced caspase-dependent apoptosis, in part by inhibiting the PI3K/AKT signaling pathway. We also found that ailanthone significantly inhibited angiogenesis and tumor growth *in vivo*, without showing obvious toxicity. However, further studies are required to identify the specific molecular targets of ailanthone and the signaling pathways affected *in vitro* and *in vivo* by treatment with ailanthone. In addition, whether ailanthone exhibits the similar effects on other cellular systems still remains to be further investigated. Nevertheless, our results shed some light on the mechanisms behind the effect of ailanthone on HCC and suggest that ailanthone could be a potential candidate for the development of novel treatment strategies for HCC.

## Methods

### Reagents

Ailanthone (purity ≥ 98%) was extracted and isolated from *Ailanthus altissima* as reported previously, and its chemical structure was identified by spectral data (Supplementary Figure)^15^. The reagents 3-(4,5-dimethylthiazol-2-yl)-2,5-diphenyltetrazolium bromide (MTT), 5,5′,6,6′-tetrachloro-1,1′,3,3′-tetraethylbenzimidazolyl-carbocyanine iodide (JC-1), propidium iodide (PI), ribonuclease A (RNase A) and 4′,6-diamidino-2-phenylindole dihydrochloride (DAPI) were supplied by Sigma (St. Louis, MO, USA). The TACS^TM^ Annexin V-FITC/PI staining assay kit was obtained from Trevigen Inc. (Gaithersburg, MD, USA). The OxiSelect^TM^ comet assay kit was purchased from Cell Biolabs (San Diego, CA, USA). Dulbecco’s modified Eagle’s medium (DMEM), fetal bovine serum (FBS) and penicillin-streptomycin (PS) were purchased from Gibco BRL (Life Technologies, NY, USA). Protease inhibitor cocktail tablets were purchased from Roche Applied Science (Mannheim, Germany). The CD31 antibody was purchased from R&D Systems (Minneapolis, MN, USA). Other antibodies were purchased from Cell Signaling Technology, Inc. (Manchester, NH, USA).

### Cell culture

The human HCC cell line Huh7 was obtained from the Chinese Academy of Sciences. The cells were maintained in a 95% air and 5% CO_2_ humidified atmosphere at 37 °C. DMEM medium supplemented with 10% FBS and 1% PS was used for routine subculturing and for all experiments.

### Cell viability assay

Cells (5 × 10^3^/well) were seeded into 96-well plates and cultured overnight. Different concentrations of ailanthone were added to each well for different times. Next, an MTT solution (5 mg/mL) was added to each well for an additional 4 h. The resulting formazan crystals were dissolved in DMSO, and the optical density was measured at 595 nm using a DTX 880 Multimode Detector (Beckman, USA).

### Colony-formation assay

Cells (5 × 10^2^/well) were seeded into 6-well plates and cultured overnight before being incubated with different concentrations of ailanthone for another 48 h. The cells were then maintained for 10 days with fresh medium. The resulting colonies were fixed with methanol at −20 °C for 30 min and were stained with 1% crystal violet for 15 min. The number of colonies >0.5 mm in diameter was counted under an inverted phase-contrast IX51 microscope (Olympus, Japan).

### DNA content analysis

Huh7 cells (3 × 10^5^/well) were seeded into 6-well plates, cultured overnight and treated with different concentrations of ailanthone for different times. The cells were fixed in 75% ethanol at −4 °C overnight, and then incubated with 50 ng/mL PI staining solution and 0.1 mg/mL RNase A in the dark for 15 min at room temperature. The DNA content of the cells was analyzed using a Guava Easy Cytometer (Guava Technologies, Millipore, Hayward, CA, USA). The data were analyzed with ModFit LT software (Becton Dickinson, CA, USA).

### Comet assay

Cells were collected after exposure to ailanthone for 48 h and were then transferred onto OxiSelect™ comet slides by mixing them with OxiSelect™ comet agarose. The cells were lysed by a 30-min incubation in 10 mM Tris, 100 mM EDTA, 2.5 M NaCl, 10% DMSO, and 1% Triton X-100. DNA unwinding was accomplished by incubation for 20 min at 4 °C under alkaline conditions (300 mM NaOH and 1 mM EDTA, pH ≥ 13). Electrophoresis was performed at 4 °C with an electrical current of 250 mA for 20 min. After electrophoresis, the slides were washed twice for 5 min each at 4 °C in a neutralizing buffer (0.4 M Tris, pH 7.5), dehydrated in 70% ethanol, stained with Vista Green DNA dye and then analyzed using a fluorescence microscope (200× magnification, Zeiss Axio Imager A2 microscope, Carl Zeiss AG, Oberkochen, Germany). At least 10 cells per sample were evaluated using MetaXpress Software to quantify tail length, tail DNA%, tail moment length and olive tail moment. The tail length is the length of the tail (in pixels); the tail DNA% is calculated as (tail DNA intensity/cell DNA intensity) ×100; the tail moment length is the length from the center of the head to the center of the tail; and the olive tail moment is calculated as the tail moment length × tail DNA%.

### Immunofluorescence

Cells were seeded onto chamber slides. After treatment with ailanthone for 48 h, the cells were fixed with 4% formaldehyde for 10 min and permeabilized with 0.1% Triton X-100 for 30 min. Next, the cells were blocked with 5% BSA and incubated with anti-p-H2AX (Ser^139^) antibody at 4 °C overnight. After rinsing with PBS, the cells were incubated with an Alexa Fluor 488-conjugated secondary antibody for 1 h and DAPI for 10 min. Images were acquired with a laser scanning confocal microscope (LSM510, Zeiss) using a 60× objective. The foci (green) and fluorescence intensity in individual cell were measured by MetaXpress Software.

### Apoptosis Assay with Annexin V-FITC/PI staining

Huh7 cells (3 × 10^5^/well) were seeded into 6-well plates and cultured overnight prior to exposure to different concentrations of ailanthone for different times. The cells were then gently collected and incubated with Annexin V-FITC/PI. According to the manufacturer’s protocol, the detection of green fluorescence from Annexin V-FITC and red fluorescence from PI was analyzed using a Guava Easy Cytometer.

### Detection of mitochondrial transmembrane potential (Δ*Ψm*)

Huh7 cells (3 × 10^5^/well) were seeded into 6-well plates and cultured overnight prior to exposure to different concentrations of ailanthone for different times. The cells were then gently collected and incubated with 10 μg/mL JC-1 in the dark for 15 min at room temperature. JC-1 fluorescence was detected using a Guava Easy Cytometer. The data were analyzed with Flow Jo 7.6 software (Tree Star, Inc. USA).

### Western blot analysis

Cells (2 × 10^6^/dish) were seeded into 100-mm culture dishes and cultured overnight before being incubated with different concentrations of ailanthone for different times. After treatment, the cells were collected and lysed in RIPA buffer containing 0.1 M PMSF, protease and phosphatase inhibitor cocktail for 30 min on ice. The lysates were centrifuged at 12,000× *g* at 4 °C for 15 min, and the supernatant was collected and stored at −80 °C. To isolate the cytosolic fraction and mitochondrial fraction, the cells were collected and lysed in cytosolic lysis buffer with protease and phosphatase inhibitors (200 μg/ml digitonin, 20 μg/ml aprotinin, 10 μg/ml pepstatin A, 50 mM NaF, 1 mM benzamidine, 2 μM EDTA, 0.1 M PMSF, 0.5 μM okadaic acid) for 30 min on ice, followed by centrifugation at 12,000× g at 4 °C for 15 min. The supernatant was collected as the cytosolic fraction. The cell pellets were further lysed in mitochondrial lysis buffer (50 mM Tris, 150 mM NaCl, 1% NP-40) with protease and phosphatase inhibitors for 30 min on ice. The lysates were then centrifuged at 12,000× g at 4 °C for 15 min. The supernatant was collected as the mitochondrial fraction. Cell lysates (40 μg) were separated by SDS-PAGE, transferred to PVDF membranes, blocked with 5% BSA and probed with a primary antibody (1:1,000) and an appropriate secondary antibody (1:2,000). Immunoreactive bands were visualized with enhanced chemiluminescent substrates using an X-ray film processor (Kodak, USA).

### Tumor xenograft in nude mice

Male nu/nu BALB/c mice (4–6 weeks old) were obtained from the Vital River Laboratory Animal Technology Co. Ltd. (Beijing, China). Huh7 cells (1 × 10^7^) were inoculated subcutaneously on the backs of nude mice and allowed to grow for approximately 14 days to reach a tumor volume of approximately 100 mm^3^. The mice were randomly divided into five groups (six mice per group). The treatment group animals were treated with ailanthone (dissolved in saline for application) at concentrations of 5 mg/kg, 10 mg/kg, or 15 mg/kg, the positive control animals were treated with 5-fluorouracil (5-Fu) at a concentration of 20 mg/kg, and the negative control group was treated with an equal volume of vehicle by intraperitoneal injection. Tumor growth was monitored by measuring the tumor size every 2 days with a digital caliper. The tumor volume was calculated using the following formula: 0.5 × *a* × *b*^2^, where *a* refers to the long diameter and *b* to the short diameter of the tumor. Body weight was monitored every 2 days throughout the treatment period. The mice were euthanized at the end of the experiment, and the tumor xenografts were removed and weighed.

### Immunohistochemical (IHC) staining

Xenograft tumor and tissue samples were fixed in 10% neutral buffered formalin, dehydrated, and embedded in paraffin. The samples were then sectioned at a 5-μm thickness and stained with hematoxylin and eosin (H&E) for histopathology under a light microscope (200× magnification). To detect apoptosis as well as levels of CDK4, CD31 and p-VEGFR2 in excised xenograft tumors, the sections were washed with PBS, blocked with 1% BSA, probed with a primary antibody (1:100), then a biotinylated secondary antibody (1:400), and then with horseradish peroxidase-conjugated streptavidin for 30 min at room temperature. The microvessel density (MVD) was evaluated by counting the number of vessels in three fields per slide, the integrated optical density (IOD) values of TUNEL, CDK4 and p-VEGFR2 at each visual field (400× magnification) were calculated by Image-Pro Plus 6.0 software (Rockville, MD, USA).

### Statistical analysis

The data are presented as the mean ± standard deviation (SD). GraphPad Prism 5.0 (Graph Pad Software, La Jolla, CA, USA) was used for statistical analysis. Statistical analysis was performed with one-way analysis of variance (ANOVA). *P* < 0.05 was considered statistically significant.

## Additional Information

**How to cite this article**: Zhuo, Z. *et al.* Ailanthone Inhibits Huh7 Cancer Cell Growth *via* Cell Cycle Arrest and Apoptosis *In Vitro* and *In Vivo*. *Sci. Rep.* **5**, 16185; doi: 10.1038/srep16185 (2015).

## Supplementary Material

Supplementary Information
