# Supplementary material for: Ailanthone Inhibits Huh7 Cancer Cell Growth via Cell Cycle Arrest and Apoptosis In Vitro and In Vivo
Source: Sci Rep. 2015 Nov 3;5:16185. doi: 10.1038/srep16185 (PMC4630794; doi:10.1038/srep16185)
Supplement: Supplementary Information [file srep16185-s1.pdf]

Ailanthone Inhibits Huh7 Cancer Cell Growth *via* Cell Cycle Arrest and Apoptosis *In Vitro* and *In Vivo*

Zhenjian Zhuo[1,*], Jianyang Hu[1,*], Xiaolin Yang[1], Minfen Chen[1], Xueping Lei[1], Lijuan Deng[1], Nan Yao[1], Qunlong Peng[1], Zhesheng Chen[2], Wencai Ye[1] & Dongmei Zhang[1]

[1]Guangdong Province Key Laboratory of Pharmacodynamic Constituents of TCM and New Drug Research, College of Pharmacy, Jinan University, Guangzhou 510632, China. [2]Department of Pharmaceutical Sciences, College of Pharmacy and Health Sciences, St. John's University, Queens, New York, United States. [*]These authors contributed equally to this work. Correspondence and requests for materials should be addressed to W.Y. (email: chyewc@gmail.com) or D.Z. (email: dmzhang701@foxmail.com)

Supplementary Information:

Identification of ailanthone by ^{1}H-NMR and ^{13}C-NMR

^{1}H-NMR (500 MHz, MeOD): δ 6.06 (1H, br s, H-3), 5.21 (1H, s, H-21), 4.62 (1H, s, H-1), 4.26 (s, 1H), 3.99 (d, J = 8.4 Hz, 1H), 3.89 (s, 1H), 3.49 (d, J = 8.4 Hz, 1H), 3.31 (s, 3H), 3.16-3.05 (m, 1H), 2.95 (s, 1H), 2.89 (t, J = 14.0 Hz, 1H), 2.84 (s, 1H), 2.69-2.59 (m, 1H), 2.25 (s, 1H), 2.13 (s, 1H), 2.02 (s, 3H), 1.20 (s, 3H). ^{13}C-NMR (125 MHz, MeOD): 84.34 (C-1), 198.78 (C-2), 126.08 (C-3), 164.97 (C-4), 46.15 (C-5), 26.66 (C-6), 79.86 (C-7), 46.40 (C-8), 48.04 (C-9), 46.40 (C-10), 110.14 (C-11), 80.89 (C-12), 146.65 (C-13), 43.31 (C-14), 35.40 (C-15), 172.25 (C-16), 22.85 (C-18), 9.98 (C-19), 72.95 (C-20), 120.21 (C-21).

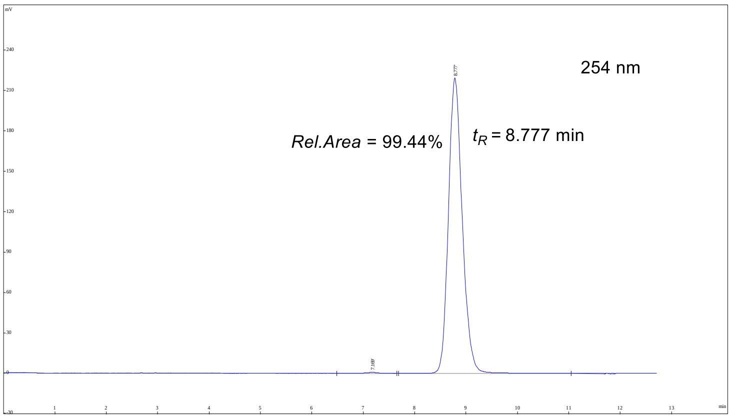

Supplemental Figure 1. Purity of ailanthone was assessed by HPLC. HPLC profiles of ailanthone under the same eluting systems (Phenomex Gemini C18 column (4.6×250 mm, 5 μm), 25% MeOH/H2O (v/v), 1.0 mL/min). Single peak indicated that

ailanthone was 98% pure.
